# A web-based intervention to promote physical activity in adolescents and young adults with cystic fibrosis: protocol for a randomized controlled trial

**DOI:** 10.1186/s12890-019-0942-3

**Published:** 2019-12-19

**Authors:** Narelle S. Cox, Beverley Eldridge, Sarah Rawlings, Julianna Dreger, Jennifer Corda, Jennifer Hauser, Brenda M. Button, Jennifer Bishop, Amanda Nichols, Anna Middleton, Nathan Ward, Tiffany Dwyer, Owen W. Tomlinson, Sarah Denford, Alan R. Barker, Craig A. Williams, Michael Kingsley, Paul O’Halloran, Anne E. Holland, On behalf of Youth Activity Unlimited – A Strategic Research Centre of the UK Cystic Fibrosis Trust

**Affiliations:** 1 Monash University, La Trobe University and Institute for Breathing and Sleep, Level 6, The Alfred Centre, 99 Commercial Road, Melbourne, Vic 3004 Australia; 2 La Trobe University, Level 4, The Alfred Centre, 99 Commercial Road, Melbourne, Vic 3004 Australia; 3 Monash Children’s Hospital Monash University and La Trobe University , 246 Clayton Rd, Clayton, Vic 3168 Australia; 4 Monash University, La Trobe University and Alfred Health, Level 6, The Alfred Centre, 99 Commercial Road, Melbourne, Vic 3004 Australia; 5 Physiotherapy Department Royal Children’s Hospital, 50 Flemington Road Parkville, Victoria, 3052 Australia; 6 Tasmanian Adult Cystic Fibrosis Unit, Royal Hobart Hospital, GPO Box 1061, Hobart, Tasmania 7000 Australia; 7 Departments of Physiotherapy and Respiratory Medicine Alfred Health and Department of Medicine, Nursing and Health Sciences, Monash University, 55 Commercial Road, Melbourne, Vic 3004 Australia; 8 Adult Cystic Fibrosis Service, Westmead Hospital, PO Box 533, Wentworthville, NSW 2145 Australia; 9 Monash Children’s Hospital/Monash Health CF Service, 246 Clayton Rd, Clayton, Victoria 3168 Australia; 10 Physiotherapy Department, Children’s Hospital at Westmead, Hawkesbury Road, Westmead, NSW 2145 Australia; 11 Physiotherapy and Cystic Fibrosis Services, 8E055.08, Royal Adelaide Hospital, Port Road, Adelaide, SA 5000 Australia; 12 Discipline of Physiotherapy, Faculty of Health Sciences, The University of Sydney, PO Box 170, Lidcombe, NSW 1825 Australia; 13 Children’s Health & Exercise Research Centre (CHERC), Sport and Health Sciences, College of Life and Environmental Sciences, St. Luke’s Campus, University of Exeter, Heavitree Road, Exeter, Devon EX1 2LU UK; 14 La Trobe Rural Health School, La Trobe University, Bendigo, Vic 3552 Australia; 15 School of Psychology and Public Health, La Trobe University, Bundoora, Vic 3086 Australia; 16 Monash University La Trobe University, Alfred Health and Institute for Breathing and Sleep, Level 6, The Alfred Centre, 99 Commercial Road, Melbourne, Vic 3004 Australia

**Keywords:** Physical fitness, Exercise, Telerehabilitation, Goal setting, Application, Online

## Abstract

**Background:**

Regular participation in physical activity by people with cystic fibrosis (CF) promotes positive clinical and health outcomes including reduced rate of decline in lung function, fewer hospitalizations and greater wellbeing. However adherence to exercise and activity programs is low, in part due to the substantial daily therapy burden for young people with CF. Strict infection control requirements limit the role of group exercise programs that are commonly used in other clinical groups. Investigation of methods to promote physical activity in this group has been limited. The Active Online Physical Activity in Cystic fibrosis Trial (ActionPACT) is an assessor-blinded, multi-centre, randomized controlled trial designed to compare the efficacy of a novel web-based program (ActivOnline) compared to usual care in promoting physical activity participation in adolescents and young adults with CF.

**Methods:**

Adolescents and young adults with CF will be recruited on discharge from hospital for a respiratory exacerbation. Participants randomized to the intervention group will have access to a web-based physical activity platform for the 12-week intervention period. ActivOnline allows users to track their physical activity, set goals, and self-monitor progress. All participants in both groups will be provided with standardised information regarding general physical activity recommendations for adolescents and young adults.

Outcomes will be assessed by a blinded assessor at baseline, after completion of the intervention, and at 3-months followup. Healthcare utilization will be assessed at 12 months from intervention completion. The primary outcome is change in moderate-to-vigorous physical activity participation measured objectively by accelerometry. Secondary outcomes include aerobic fitness, health-related quality of life, anxiety and depression and sleep quality.

**Discussion:**

This trial will establish whether a web-based application can improve physical activity participation more effectively than usual care in the period following hospitalization for a respiratory exacerbation. The web-based application under investigation can be made readily and widely available to all individuals with CF, to support physical activity and exercise participation at a time and location of the user’s choosing, regardless of microbiological status.

**Trial registration:**

Clinical trial registered on July 13, 2017 with the Australian and New Zealand Clinical Trials Register at (ACTRN12617001009303).

## Background

Cystic Fibrosis (CF) is a complex, multi-system inherited disorder. This progressive disorder is commonly characterised by chronic suppurative lung disease, including bronchiectasis, progressing to respiratory failure [1]. People with CF require repeated admission to hospital to treat respiratory complications; have poor quality of life and life expectancy; experience reduced aerobic fitness and physical functioning, and increased prevalence of anxiety and depression [2]. Higher levels of physical activity participation and aerobic fitness have been associated with improved health outcomes, in particular a slower decline in lung function [3], reduced need for hospitalization [4], and improved prognosis [5] in both children and adults with CF.

Regular participation in moderate-to-vigorous intensity physical activity (MVPA) has numerous health benefits including improved aerobic fitness and bone density, and reduced risk of depression [6]. Regular exercise and physical activity are recommended in CF treatment guidelines [2], to improve aerobic fitness; relieve breathlessness; and positively influence bone accretion, blood glucose control and clearance of pulmonary secretions [7, 8]. Despite the benefits of regular physical activity participation, people with CF demonstrate poor uptake and adherence to programs designed to augment physical activity and exercise [9]. This problem is compounded by the limited range of methods trialled to date to increase physical activity participation by people with CF [10].

Few strategies have been applied in the promotion of physical activity to people with CF. A Cochrane Review of strategies to promote physical activity participation by people with CF identified several studies which employed various forms of exercise training to improve physical activity, in mostly young people with mild CF lung disease [3, 10–13]. No study in the review investigated the effect of strategies such as motivational interviewing or the use of technology or telemedicine in promoting physical activity participation by people with CF. There is limited evidence that interventions of medium-longer term duration, with a requirement for self-directed participation, may be more effective than short-term, supervised training in improving physical activity participation [10].

Technology and telemedicine applications have the potential to create new ways to promote and support physical activity and exercise participation in this population, and are feasible and acceptable to people with CF [14, 15]. Importantly, no physical activity intervention study in people with CF to date has focussed on the period immediately post hospitalization. Objectively measured physical activity levels in adolescents and young adults with CF have been found to decline by over 50% in the first one month following hospital discharge [4]. In other populations with chronic respiratory disease, particularly people with chronic obstructive pulmonary disease (COPD), failure to recover physical activity levels in the month following hospital discharge is associated with an increased likelihood of hospital readmission [16]. Whether a web-based intervention to promote physical activity can effectively improve activity participation, and delay time to next admission, in young people with CF is unknown.

This paper describes the protocol for a randomized controlled trial testing a web-based application to promote physical activity in adolescents and young adults with CF. The aims of the trial are to: 1) investigate the effect of a web-based application (ActivOnline) to promote physical activity participation in young people with CF; 2) assess the effect of a technology based intervention (ActivOnline), in the period immediately following hospital discharge on aerobic fitness, lung function, quality of life, anxiety and depression, sleep quality, and healthcare utilisation in young people with CF. We hypothesise that the web-based intervention will improve uptake and participation in physical activity by young people with CF following a hospital admission for a respiratory exacerbation compared to usual care; that increased physical activity participation will lead to improvements in exercise capacity, lung function, quality of life, anxiety and depression, and sleep quality; and that healthcare utilization will be reduced in the intervention group over 12 months.

## Methods

### Design

A randomized, controlled, assessor-blinded trial will be conducted at eight Australian sites (Alfred Health, Monash Health and Royal Children’s Hospital, Victoria; Royal Hobart Hospital, Tasmania; Royal Prince Alfred Hospital, Westmead Hospital and Children’s Hospital at Westmead, New South Wales; Royal Adelaide Hospital, South Australia). The Human Research Ethics Committee at Alfred Health approved the study for all sites, and local governance approvals were obtained from all participating sites. The trial was registered at www.anzctr.org.au (ACTRN12617001009303) on July 13, 2017. This trial protocol employs our established methods with respect to randomization procedures, data integrity and management, trial safety monitoring and managing participant withdrawals [17].

### Participants and recruitment

Potential participants will be all individuals with CF admitted to hospital for a respiratory cause at the participating sites. To be eligible for inclusion participants will: 1) have a confirmed diagnosis of CF; 2) be aged 12–35 years (inclusive); and 3) have access to the internet via a computer or mobile device. Potential participants will be excluded if they: 1) have a severe co-morbidity limiting mobilisation or physical activity participation (e.g. orthopedic, cardiac or neurological condition); 2) have been the recipient of a lung transplant; 3) are pregnant; or 4) they (or their parents) are unable to provide informed consent.

### Randomisation

Participants will be randomly allocated (1:1) to ‘usual care’ or the ‘ActivOnline’ intervention. A computer-generated, block randomization scheme will be used with stratification for 1) site of recruitment – to allow for differences in local treatment practices including those related to usual prescription of exercise, and 2) whether or not the participant is enrolled in fulltime schooling (primary or secondary versus not in fulltime schooling) as the time of transition from secondary school to university or the workforce is a known time for decline in physical activity participation [18].

Sequence generation will be performed by an individual who is independent of the research team and randomization will occur using an online database. The randomization sequence will be concealed from investigators. Participants will be allocated to groups after completion of one week of physical activity monitoring immediately following discharge from hospital. Participants will not be blind to the intervention, however all outcomes will be measured by an independent assessor blind to group allocation. The flow of participants through the study will be reported according to the recommendations of the Consolidated Standard of Reporting Trials (CONSORT) [19].

### Interventions

#### Usual care

Physical activity and exercise is routinely advised for all patients with CF [20, 21]. All participants will be provided with age-appropriate information regarding recommended guidelines for physical activity participation. Participants will be referred to a free online resource (http://www.nhs.uk/Livewell/fitness/Pages/physical-activity-guidelines-for-young-people.aspx) containing guidelines and information regarding amount and intensity of daily physical activity participation [6]. Physical activities which involve the use of large muscle groups continuously will be encouraged [3], as will the preferred activities of the participants [11].

#### ActivOnline intervention

Participants randomized to the active intervention group will be given individualized access to the ActivOnline program (www.activonline.com.au) and encouraged to use this for the 12-week intervention period, to track their physical activity, set goals, and self-monitor progress. This will be in addition to usual care.

**Fig. 1:**
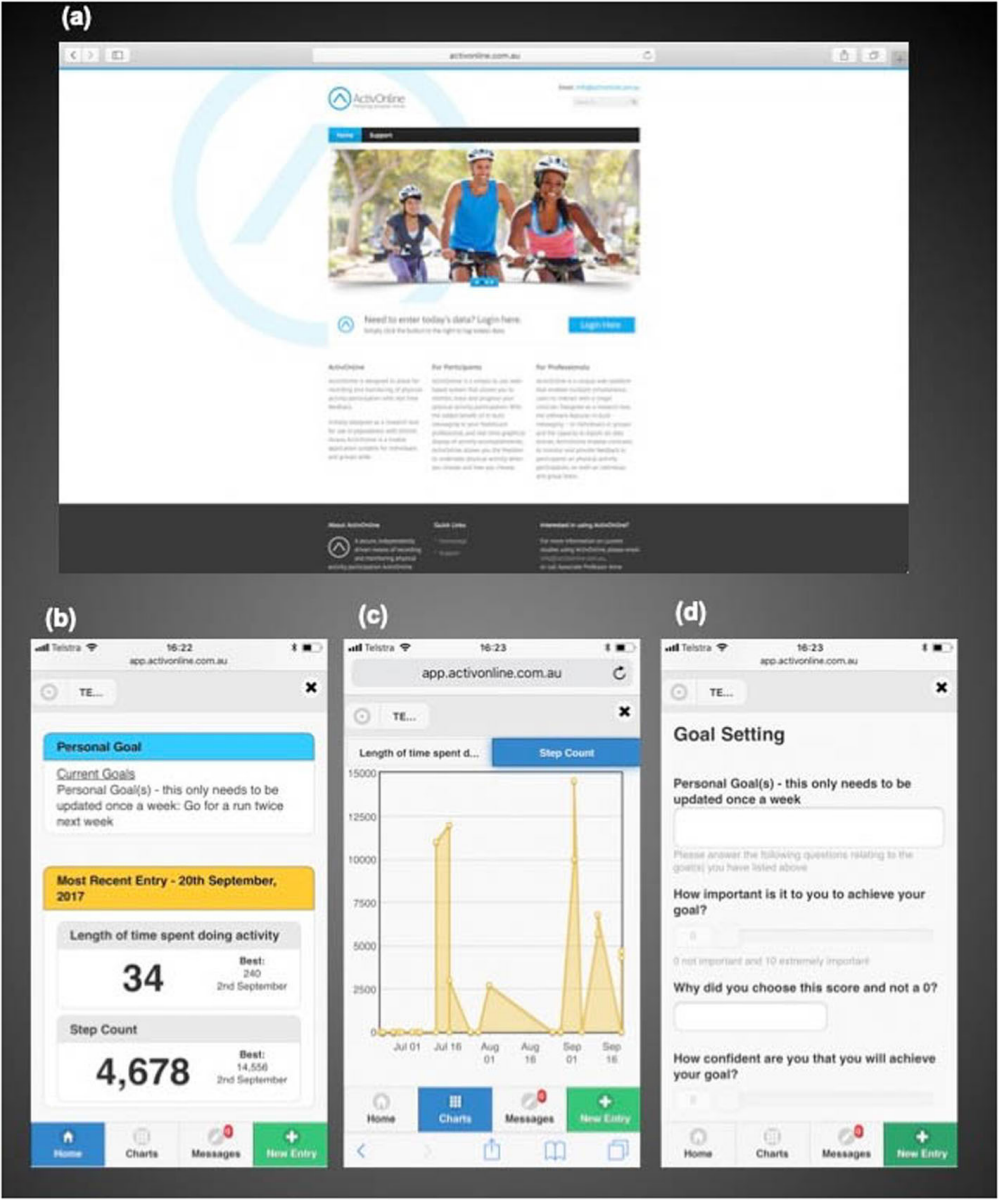
ActivOnline. **a**: ActivOnline website homepage; **b**-**d**: ActivOnline data entry portal – data entry screen (**b**); real time graphical display of data (**c**); goal setting (**d**). The image depicted in (**a**) is legally sourced royalty free stock photo from Adobe Stock

ActivOnline uses principles of motivational interviewing and cognitive behavioural strategies, with the aim of increasing opportunity and motivation for physical activity participation. It provides a secure portal for recording and reviewing physical activity and exercise participation details. ActivOnline is a mobile platform accessible from any internet browser across a variety of devices including tablets and smartphones. When logging into ActivOnline participants will be prompted to set weekly exercise and physical activity goals, as well as to record details of their physical activity or exercise sessions including total time and step count. To support recording of daily step count participants may use their own activity tracker (e.g. Fitbit) or mobile telephone. A pedometer (Yamax digiwalker SW500, Yamasa Tokei Keiki Co., Ltd., Tokyo, Japan) will be provided to participants on request. Data entered into ActivOnline are displayed in numerical and graphical form to allow visualization of progress over time (see Fig. 1). Participants can choose the frequency of use of ActivOnline, as data can be entered retrospectively. If no activity has been logged for three days, a standardized alert message will be issued by the ActivOnline program and emailed to the participant. Participants in the intervention group will also be able to communicate with research clinicians directly via the messaging system contained within ActivOnline about the trial or their clinical status, should they require. The number and nature of contacts via the messaging system will be recorded.

### Outcome measures

Demographic details of age, gender, body mass index (BMI) and lung function will be collected at baseline, prior to hospital discharge. Details pertaining to CF genotype, age at diagnosis and status for pancreatic insufficiency will be obtained from the medical record. Frequency of access to the ActivOnline program and number of exercise sessions recorded will be extracted from ActivOnline.

**Fig. 2:**
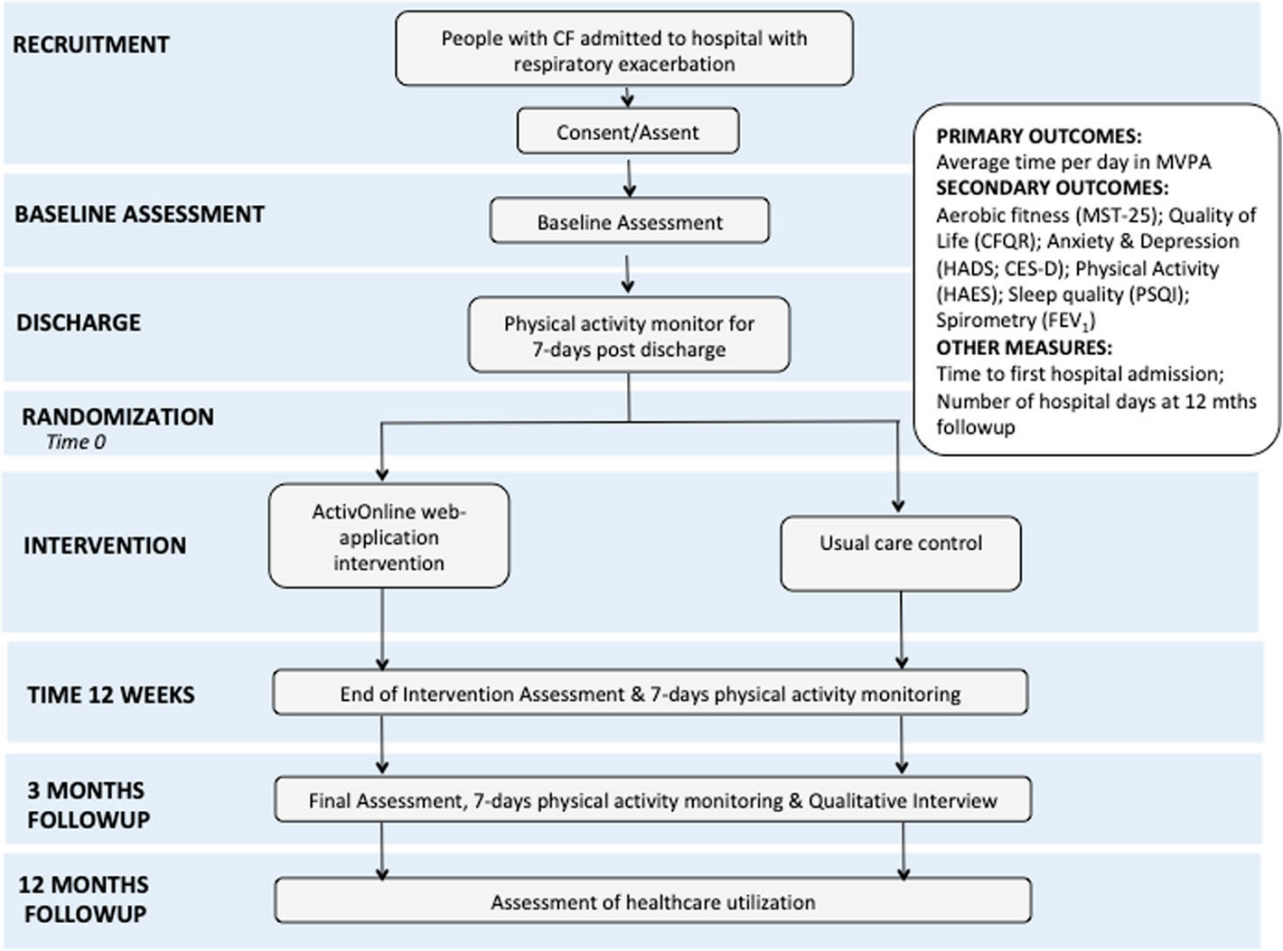
Participant schedule. MVPA = moderate-vigorous physical activity; MST-25 = Modified shuttle test (25 levels); CFQR = Cystic Fibrosis quality of life questionnaire – revised; HADS = Hospital anxiety and depression scale; CES-D = Centre for epidemiological studies depression scale; HAES = Habitual activity estimation scale; PSQI = Pittsburgh Sleep Quality Index; FEV_1_ = Forced expiratory volume in one second

Participants will undertake assessment of clinical outcome measures at baseline, end intervention and after 3 months follow-up (Fig. 2). The following measures will be recorded:

#### Primary outcome

The primary outcome will be time spent in moderate-vigorous physical activity (MVPA) as measured objectively using accelerometry. The intensity of physical activity will be monitored using a wrist-worn accelerometer provided to participants for seven days (Actigraph Link; Actigraph LLC, Pensacola, FL 32502 USA). This triaxial accelerometer is validated for the assessment of free-living activity and is a recommended device for the objective assessment of physical activity in individuals with CF [22]. The a priori definition for activity monitoring data to be included in the final analysis is a minimum of three days [23], for ≥10 h of valid wear time in each day [4]. Average time (minutes) per day spent in MVPA will be reported.

Accelerometers will be initialized (sampling rate 100 Hz) and downloaded using the ActiLife software (v6.10.4; Acti-Graph, Pensacola, Fl, USA). Wear-time will be established using ActiLife parameters such that a period of at least 90 min of consecutive activity counts of zero, with a spike threshold of two minutes and 100 counts per minute, will define non-wear time [24]. Pre-specified cut-points will be used to determine time spent in at least moderate intensity activity, with activity intensity thresholds determined by participant age [25, 26].

#### Secondary outcomes

- Self-reported physical activity participation will also be assessed using the Habitual Activity Estimation Scale (HAES), a validated subjective measure of physical activity participation in young people with CF [27]. Two measures of physical activity participation will allow for comparison between patient perception of activity intensity and objectively measured data.
- Aerobic fitness will be assessed using the Modified Shuttle Test (25 level version; MST-25) [28], an extension of a field test of aerobic capacity which is a reliable and valid measure of exercise capacity in adults and children with CF [29–31]. The MST-25 is a symptom-limited estimated assessment of peak aerobic capacity with distance covered the outcome of interest. It requires participants to walk, or run as necessary, around two markers over a 10-m course in time with a pre-recorded audio signal. Each level of the test lasts for one minute, with the speed increasing by 0.61 km/hr each minute. Participants cease the test when they feel they are no longer able to continue or when they fail to meet the course marker on two consecutive shuttles [29].
- Assessment of the reasons underlying participants decision to engage, or not engage, in physical exercise will be undertaken using the Behavioural Regulation in Exercise Questionnaire (BREQ-2) [32]. The BREQ-2 has been used in young people [33] and adults [34] with a variety of chronic medical and psychological conditions.
- Spirometry measures of forced expiratory volume in one second (FEV_1_) and forced vital capacity (FVC) will be reported. Spirometry measures will be conducted according to American Thoracic Society/ European Respiratory Society (ATS/ERS) standard procedures [35].
- Health-related quality of life (HRQoL) will be assessed using the revised edition of the Cystic Fibrosis Questionnaire (CFQ-R) [36]. The CFQ-R is a valid and reliable tool that provides a disease-specific measure of HRQoL [36].
- Anxiety and depression will be evaluated using the Hospital Anxiety and Depression Scale (HADS) [37] and the Center for Epidemiologic Studies -Depression (CES-D) scale [38]. These scales have been used in establishing the prevalence of anxiety and depression in young people and adults with CF [39].
- As exercise can positively impact both sleep and health outcomes, participants will complete the Pittsburgh Sleep Quality Index (PSQI) [40] self-report measure of sleep quality. The PSQI has been validated widely, and previously used in children and young adults with CF [41].
- Healthcare utilization will be documented from the medical record. Time to next hospital admission and the number of hospital days up to 12 months following completion of the intervention period will be reported.

### Analysis

#### Sample size

To detect a difference of 20-min per day in MVPA participation between ActivOnline and control groups following the intervention period, a total of 56 participants will be required. This sample size was based on physical activity participation measured post hospitalisation in our population of young adults with CF [4] and assumes a standard deviation of 26 [4] with a power of 80% and a significance level of *p* < 0.05. It was planned to randomize a total of 75 participants to allow for 25% attrition. Over the first 18 months of recruitment there was higher than anticipated attrition for the primary outcome. It was decided to extend recruitment beyond the initial target of 75 to account for this attrition. The original trial registration incorrectly stated a different number of participants (incorrect total 150). This was due to an error in interpreting the sample size calculation. The correct number of participants required (total for both groups combined) is *n* = 56, with *n* = 19 to allow for 25% attrition, giving a correct total sample size of *n* = 75. In our original registration we had incorrectly assumed that *n* = 56 were required in each group.

#### Analysis

Continuous variables will be analysed by fitting linear mixed models, controlling for recruitment centre and baseline values as required. The proportion of participants who achieve age recommended physical activity will be compared between groups using a chi-squared test. Time to hospital admission will be evaluated using Kaplan-Meier curves and Cox proportional hazards modeling. All data will be analysed by intention-to-treat. Alpha will be set at 0.05.

#### Data integrity and management

Data will be stored on a purpose-built online database (www.adeptrs.com), with encryption, password-protection and restricted access. No identifying information will be stored in the online database.

#### Withdrawal

A participant will be considered to have withdrawn from the study when consent is revoked. If this occurs, no further assessments will be performed. Participants will be informed that data collected up to the time of withdrawal will form part of the study results unless permission is expressly declined. Withdrawn participants will not be replaced. Protocol violations will not constitute grounds for withdrawal. Study withdrawal will not have any impact on care provided by any of the participating sites.

### Monitoring

The trial will be monitored by an independent Data Safety Monitoring Board (DSMB) comprising a respiratory physician and two clinical research physiotherapists, with consultation with a statistician as required. The DSMB will review data relating to the primary outcome (MVPA participation), as well as quality of life and safety. Data will be presented to the DSMB in a blinded fashion. The DSMB will initially review data at a time six months from the commencement of recruitment, and six monthly thereafter. Any serious adverse events will be notified immediately to the overseeing ethics committee (Alfred Health) and the relevant site governance committee, as well as to the DSMB. If there are concerns about the safety of participants, the DSMB will make a recommendation to the trial steering committee about continuing, stopping, or modifying the trial.

## Discussion

Regular physical activity is a key recommended component of international care guidelines for individuals with CF [20, 21], yet only limited methods for supporting patients to adhere to this component of their therapeutic regimen have been investigated. This study will compare the effects of a web-based platform which enables activity tracking, self-monitoring and goal setting, compared to usual care on clinical outcomes and healthcare utilization in adolescents and young adults with CF.

Internationally, annual medical expenditure on individuals with CF is 22 times greater than those without CF [42]. Physical activity participation is a low-cost treatment strategy that has the potential to reduce the impact and progression of chronic lung disease in CF, and associated healthcare expenditure. To date, few strategies outside of exercise training programs have been explored as means to promote daily physical activity participation by people with CF [10]. The physical activity promotion strategy under investigation addresses key therapy delivery issues associated with treatment timing and infection control. By providing opportunity for goal-setting and self-monitoring, at a time and place convenient to the patient, challenges of adherence to exercise programs and maintenance of activity following hospitalisation might be reduced.

## Trial status

Recruitment commenced in September 2017 and remains ongoing.

## Data Availability

Not applicable. Details relating to planned data availability are available in the clinical trials registration at www.ANZCTR.org.au.

## Abbreviations

BREQ2: behavioural regulation in exercise questionnaire (version 2)
CES-D: center for epidemiologic studies -depression scale
CF: cystic fibrosis
CFQR: cystic fibrosis questionnaire – revised
DSMB: data safety monitoring board
FEV_1_: forced expiratory volume in one second
HADS: hospital anxiety and depression scale
HAES: habitual activity estimation scale
HRQoL: health related quality of life
MST-25: modified shuttle test (25 level)
MVPA: moderate-vigorous physical activity
PSQI: Pittsburgh sleep quality index
